# 1,4-Dimeth­oxy-2,5-bis­{2-[4-(trifluoro­meth­yl)phen­yl]ethyn­yl}benzene

**DOI:** 10.1107/S160053681102099X

**Published:** 2011-06-11

**Authors:** Baohai Zhao, Jimao Lin, Cuihua Zhao, Ziying Wang

**Affiliations:** a School of Chemistry and Chemical Engineering, Shandong University, Jinan 250100, People’s Republic of China; b Department of Chemistry and Environmental Science, Taishan University, Taian 271021, Shandong, People’s Republic of China

## Abstract

The asymmetric unit of the title compound, C_26_H_16_F_6_O_2_, contains one half of the mol­ecule situated on an inversion centre. In the rod-like mol­ecule, the two terminal benzene rings form a dihedral angle of 71.9 (1)° with the central benzene ring. The trifluoro­methyl group is rotationally disordered over two orientations in a 0.53 (1):0.47 (1) ratio. The crystal packing exhibits no classical inter­molecular inter­actions.

## Related literature

For applications and details of the synthesis of (aryl­ene)­ethynylene derivatives, see: Dirk *et al.* (2001 ▶); Miljanić *et al.* (2005 ▶); Morin *et al.* (2007 ▶). For the crystal structure of a related 1,4-bis­(*p*-tolyl­ethyn­yl)benzene, see: Filatov & Petrukhina (2005 ▶).
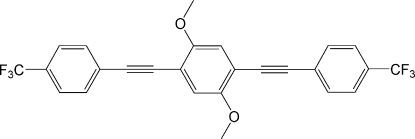


## Experimental

### 

#### Crystal data

C_26_H_16_F_6_O_2_
*M* *_r_* = 474.39
Monoclinic, 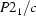

*a* = 11.1473 (4) Å
*b* = 13.0795 (6) Å
*c* = 7.5875 (4) Å
β = 97.467 (3)°
*V* = 1096.88 (9) Å^3^
*Z* = 2
Mo *K*α radiation
μ = 0.13 mm^−1^
*T* = 293 K
0.22 × 0.20 × 0.19 mm

#### Data collection

Bruker APEXII CCD diffractometer
Absorption correction: multi-scan (*SADABS*; Bruker, 2007 ▶) *T*_min_ = 0.973, *T*_max_ = 0.977
9899 measured reflections
2484 independent reflections
1753 reflections with *I* > 2σ(*I*)
*R*_int_ = 0.022

#### Refinement

*R*[*F* ^2^ > 2σ(*F* ^2^)] = 0.042
*wR*(*F* ^2^) = 0.117
*S* = 1.04
2484 reflections
184 parameters
30 restraints
H-atom parameters constrained
Δρ_max_ = 0.16 e Å^−3^
Δρ_min_ = −0.21 e Å^−3^

### 

Data collection: *APEX2* (Bruker, 2007 ▶); cell refinement: *SAINT* (Bruker, 2007 ▶); data reduction: *SAINT*; program(s) used to solve structure: *SHELXS97* (Sheldrick, 2008 ▶); program(s) used to refine structure: *SHELXL97* (Sheldrick, 2008 ▶); molecular graphics: *XP* in *SHELXTL* (Sheldrick, 2008 ▶); software used to prepare material for publication: *SHELXTL*.

## Supplementary Material

Crystal structure: contains datablock(s) I, global. DOI: 10.1107/S160053681102099X/cv5093sup1.cif

Structure factors: contains datablock(s) I. DOI: 10.1107/S160053681102099X/cv5093Isup2.hkl

Supplementary material file. DOI: 10.1107/S160053681102099X/cv5093Isup3.cml

Additional supplementary materials:  crystallographic information; 3D view; checkCIF report

## Figures and Tables

**Fig. 1.**
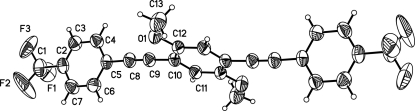
The molecular structure of (I) showing the atom-numbering scheme and 50% probabilty displacement ellipsoids. Unlabelled atoms are related with the labelled ones by symmetry operation (-x, 2 - y, 2 - z). For the disordered F atoms, only major parts are shown.

## supplementary crystallographic information

### Comment

Recently, the synthesis and applications of new aryleneethynylene derivatives were reported (Dirk *et al.*, 2001; Miljanić *et al.*, 2005; Morin *et al.*, 2007). To make our own contribution in this field of material science, herewith we report the synthesis and crystal structure of the title compound, (I), which can be used as luminescent material.

In (I) (Fig. 1), all bond lengths and angles are normal and correspond to those reported for 1,4-bis(*p*-tolylethynyl)benzene (Filatov & Petrukhina, 2005). The asymmetric unit of (I) contains a half of the rod-like molecule. The centroid of the central benzene ring is situated on an inversion centre. The central benzene ring and C2–C7 ring form a dihedral angle of 71.9 (1)°. The crystal packing exhibits no classical intermolecular interactions.

### Experimental

1,4-Dimethoxy-2,5-diethynylbenzene (93 mg, 0.5 mmol), Pd(PPh_3_)_2_Cl_2_ (17.5 mg)and CuI (9.5 mg) were added to triethylamine (3 ml) and tetrahydrofuran (9 ml)in a Schlenk flask under N_2_ atmosphere. The mixture was stirred at room temperature overnight. Then the solution was cooled to room temperature and the solvent was removed in vacuum. CH_2_Cl_2_ (15 ml) was added and the suspension was filtered. The filtrate was washed with HCl (1 mol l^-1^), ammonium chloride solution and water. Then organic phase was dried with MgSO_4_ and concentrated. The crude product was purified by column chromatography on silica gel to afford the title compound (185.3 mg, 78%). Crystals suitable for X-ray structure analysis were obtained by slowly evaporating dichloromethane solution of the title compound at room temperature.

### Refinement

All the H atoms were treated as riding atoms in geometrically idealized positions (C—H 0.93–0.96 Å), with U_iso_(H) = 1.2–1.5U_eq_(C). Trifluoromethyl fragment was treated as rotationally disordered over two orientations with the refined occupancies of 0.53 (1):0.47 (1), respectively.

### Crystal data

**Table:**

C_26_H_16_F_6_O_2_	*F*(000) = 484
*M**_r_* = 474.39	*D*_x_ = 1.436 Mg m^−^^3^
Monoclinic, *P*2_1_/*c*	Mo *K*α radiation, λ = 0.71073 Å
*a* = 11.1473 (4) Å	θ = 2.4–27.4°
*b* = 13.0795 (6) Å	µ = 0.13 mm^−^^1^
*c* = 7.5875 (4) Å	*T* = 293 K
β = 97.467 (3)°	Block, colourless
*V* = 1096.88 (9) Å^3^	0.22 × 0.20 × 0.19 mm
*Z* = 2	

### Data collection

**Table:**

Bruker APEXII CCD diffractometer	2484 independent reflections
Radiation source: fine-focus sealed tube	1753 reflections with *I* > 2σ(*I*)
graphite	*R*_int_ = 0.022
φ and ω scans	θ_max_ = 27.4°, θ_min_ = 2.4°
Absorption correction: multi-scan (*SADABS*; Bruker, 2007)	*h* = −14→14
*T*_min_ = 0.973, *T*_max_ = 0.977	*k* = −15→16
9899 measured reflections	*l* = −9→9

### Refinement

**Table:**

Refinement on *F*^2^	Secondary atom site location: difference Fourier map
Least-squares matrix: full	Hydrogen site location: inferred from neighbouring sites
*R*[*F*^2^ > 2σ(*F*^2^)] = 0.042	H-atom parameters constrained
*wR*(*F*^2^) = 0.117	*w* = 1/[σ^2^(*F*_o_^2^) + (0.0488*P*)^2^ + 0.235*P*] where *P* = (*F*_o_^2^ + 2*F*_c_^2^)/3
*S* = 1.04	(Δ/σ)_max_ < 0.001
2484 reflections	Δρ_max_ = 0.16 e Å^−^^3^
184 parameters	Δρ_min_ = −0.21 e Å^−^^3^
30 restraints	Extinction correction: *SHELXL97* (Sheldrick, 2008), Fc^*^=kFc[1+0.001xFc^2^λ^3^/sin(2θ)]^-1/4^
Primary atom site location: structure-invariant direct methods	Extinction coefficient: 0.011 (3)

### Special details

**Table:**

Geometry. All e.s.d.'s (except the e.s.d. in the dihedral angle between two l.s. planes) are estimated using the full covariance matrix. The cell e.s.d.'s are taken into account individually in the estimation of e.s.d.'s in distances, angles and torsion angles; correlations between e.s.d.'s in cell parameters are only used when they are defined by crystal symmetry. An approximate (isotropic) treatment of cell e.s.d.'s is used for estimating e.s.d.'s involving l.s. planes.
Refinement. Refinement of *F*^2^ against ALL reflections. The weighted *R*-factor *wR* and goodness of fit *S* are based on *F*^2^, conventional *R*-factors *R* are based on *F*, with *F* set to zero for negative *F*^2^. The threshold expression of *F*^2^ > σ(*F*^2^) is used only for calculating *R*-factors(gt) *etc*. and is not relevant to the choice of reflections for refinement. *R*-factors based on *F*^2^ are statistically about twice as large as those based on *F*, and *R*-factors based on ALL data will be even larger.

### Fractional atomic coordinates and isotropic or equivalent isotropic displacement parameters (Å^2^)

**Table:**

	*x*	*y*	*z*	*U*_iso_*/*U*_eq_	Occ. (<1)
C1	0.75647 (18)	0.83209 (17)	0.5592 (3)	0.0718 (6)	
C2	0.63855 (14)	0.85402 (14)	0.6275 (2)	0.0527 (4)	
C3	0.54917 (16)	0.78099 (15)	0.6153 (3)	0.0588 (5)	
H3	0.5617	0.7178	0.5647	0.071*	
C4	0.44076 (15)	0.80134 (14)	0.6781 (2)	0.0562 (5)	
H4	0.3805	0.7518	0.6697	0.067*	
C5	0.42165 (13)	0.89548 (14)	0.7537 (2)	0.0473 (4)	
C6	0.51220 (16)	0.96868 (14)	0.7635 (3)	0.0589 (5)	
H6	0.4999	1.0322	0.8131	0.071*	
C7	0.62038 (16)	0.94824 (15)	0.7003 (3)	0.0608 (5)	
H7	0.6806	0.9978	0.7069	0.073*	
C8	0.31058 (14)	0.91755 (14)	0.8243 (2)	0.0525 (4)	
C9	0.22045 (14)	0.93931 (13)	0.8846 (2)	0.0497 (4)	
C10	0.10877 (13)	0.96910 (12)	0.9454 (2)	0.0452 (4)	
C11	0.03456 (14)	1.03921 (12)	0.8455 (2)	0.0477 (4)	
H11	0.0584	1.0656	0.7417	0.057*	
C12	0.07381 (13)	0.92971 (12)	1.1021 (2)	0.0455 (4)	
C13	0.1199 (2)	0.82346 (18)	1.3576 (3)	0.0769 (6)	
H13A	0.1109	0.8791	1.4372	0.115*	
H13B	0.1823	0.7782	1.4105	0.115*	
H13C	0.0449	0.7867	1.3348	0.115*	
O1	0.15207 (10)	0.86246 (10)	1.19450 (17)	0.0605 (4)	
F1	0.7461 (6)	0.8553 (4)	0.3877 (5)	0.0928 (16)	0.530 (10)
F2	0.8484 (5)	0.8832 (9)	0.6296 (14)	0.183 (5)	0.530 (10)
F3	0.7807 (6)	0.7351 (3)	0.5523 (9)	0.125 (3)	0.530 (10)
F1'	0.7695 (6)	0.7396 (4)	0.5079 (11)	0.136 (4)	0.470 (10)
F2'	0.8458 (4)	0.8398 (6)	0.6905 (7)	0.0953 (18)	0.470 (10)
F3'	0.7830 (9)	0.8979 (10)	0.4453 (17)	0.205 (6)	0.470 (10)

### Atomic displacement parameters (Å^2^)

**Table:**

	*U*^11^	*U*^22^	*U*^33^	*U*^12^	*U*^13^	*U*^23^
C1	0.0550 (12)	0.0973 (17)	0.0678 (14)	0.0107 (11)	0.0252 (10)	−0.0029 (13)
C2	0.0413 (8)	0.0727 (12)	0.0462 (9)	0.0070 (8)	0.0135 (7)	−0.0022 (8)
C3	0.0545 (10)	0.0638 (12)	0.0603 (11)	0.0052 (8)	0.0152 (8)	−0.0127 (9)
C4	0.0465 (9)	0.0623 (11)	0.0613 (11)	−0.0033 (8)	0.0128 (8)	−0.0070 (9)
C5	0.0374 (8)	0.0606 (10)	0.0454 (9)	0.0062 (7)	0.0118 (7)	0.0025 (8)
C6	0.0543 (10)	0.0562 (11)	0.0703 (12)	0.0008 (8)	0.0239 (9)	−0.0095 (9)
C7	0.0468 (9)	0.0697 (12)	0.0696 (12)	−0.0083 (8)	0.0213 (9)	−0.0073 (10)
C8	0.0438 (9)	0.0616 (11)	0.0542 (10)	0.0045 (8)	0.0146 (7)	0.0039 (8)
C9	0.0409 (8)	0.0552 (10)	0.0552 (10)	0.0014 (7)	0.0142 (7)	0.0028 (8)
C10	0.0354 (7)	0.0494 (9)	0.0531 (9)	−0.0011 (7)	0.0144 (7)	−0.0027 (7)
C11	0.0422 (8)	0.0532 (10)	0.0503 (9)	−0.0016 (7)	0.0162 (7)	0.0039 (8)
C12	0.0379 (8)	0.0464 (9)	0.0532 (10)	0.0014 (6)	0.0099 (7)	0.0029 (7)
C13	0.0759 (13)	0.0846 (15)	0.0732 (14)	0.0208 (11)	0.0207 (11)	0.0317 (12)
O1	0.0510 (7)	0.0679 (8)	0.0653 (8)	0.0144 (6)	0.0170 (6)	0.0167 (6)
F1	0.089 (3)	0.120 (3)	0.081 (2)	0.005 (2)	0.0551 (18)	0.006 (2)
F2	0.056 (3)	0.278 (10)	0.228 (8)	−0.065 (5)	0.068 (4)	−0.175 (8)
F3	0.119 (4)	0.109 (4)	0.168 (5)	0.074 (3)	0.095 (4)	0.067 (4)
F1'	0.071 (3)	0.180 (8)	0.163 (5)	0.005 (3)	0.031 (3)	−0.118 (6)
F2'	0.0321 (19)	0.142 (4)	0.115 (3)	0.0013 (19)	0.0200 (17)	0.000 (3)
F3'	0.142 (8)	0.247 (10)	0.260 (10)	0.105 (7)	0.157 (8)	0.190 (9)

### Geometric parameters (Å, °)

**Table:**

C1—F2	1.281 (4)	C6—C7	1.380 (2)
C1—F3'	1.281 (4)	C6—H6	0.9300
C1—F1'	1.285 (4)	C7—H7	0.9300
C1—F3	1.300 (4)	C8—C9	1.191 (2)
C1—F2'	1.318 (4)	C9—C10	1.437 (2)
C1—F1	1.327 (4)	C10—C11	1.391 (2)
C1—C2	1.502 (2)	C10—C12	1.397 (2)
C2—C3	1.375 (3)	C11—C12^i^	1.381 (2)
C2—C7	1.376 (3)	C11—H11	0.9300
C3—C4	1.381 (2)	C12—O1	1.3664 (19)
C3—H3	0.9300	C12—C11^i^	1.381 (2)
C4—C5	1.386 (2)	C13—O1	1.427 (2)
C4—H4	0.9300	C13—H13A	0.9600
C5—C6	1.386 (2)	C13—H13B	0.9600
C5—C8	1.441 (2)	C13—H13C	0.9600
			
F2—C1—F3'	71.9 (5)	C5—C4—H4	119.9
F2—C1—F1'	120.1 (5)	C6—C5—C4	119.06 (14)
F3'—C1—F1'	112.5 (6)	C6—C5—C8	119.78 (16)
F2—C1—F3	111.5 (5)	C4—C5—C8	121.15 (16)
F3'—C1—F3	124.1 (6)	C7—C6—C5	120.70 (17)
F1'—C1—F3	15.5 (5)	C7—C6—H6	119.6
F2—C1—F2'	32.8 (6)	C5—C6—H6	119.6
F3'—C1—F2'	103.9 (7)	C2—C7—C6	119.57 (17)
F1'—C1—F2'	101.3 (4)	C2—C7—H7	120.2
F3—C1—F2'	87.9 (5)	C6—C7—H7	120.2
F2—C1—F1	104.7 (6)	C9—C8—C5	177.5 (2)
F3'—C1—F1	35.2 (8)	C8—C9—C10	175.89 (19)
F1'—C1—F1	85.2 (4)	C11—C10—C12	119.64 (13)
F3—C1—F1	100.1 (4)	C11—C10—C9	118.86 (14)
F2'—C1—F1	133.2 (3)	C12—C10—C9	121.50 (15)
F2—C1—C2	116.4 (3)	C12^i^—C11—C10	121.18 (15)
F3'—C1—C2	113.2 (3)	C12^i^—C11—H11	119.4
F1'—C1—C2	115.0 (4)	C10—C11—H11	119.4
F3—C1—C2	113.4 (3)	O1—C12—C11^i^	124.45 (15)
F2'—C1—C2	109.5 (3)	O1—C12—C10	116.37 (13)
F1—C1—C2	109.1 (3)	C11^i^—C12—C10	119.18 (15)
C3—C2—C7	120.41 (15)	O1—C13—H13A	109.5
C3—C2—C1	120.20 (17)	O1—C13—H13B	109.5
C7—C2—C1	119.38 (17)	H13A—C13—H13B	109.5
C2—C3—C4	120.11 (17)	O1—C13—H13C	109.5
C2—C3—H3	119.9	H13A—C13—H13C	109.5
C4—C3—H3	119.9	H13B—C13—H13C	109.5
C3—C4—C5	120.14 (17)	C12—O1—C13	117.37 (13)
C3—C4—H4	119.9		
			
F2—C1—C2—C3	−154.8 (8)	C8—C5—C6—C7	178.50 (18)
F3'—C1—C2—C3	124.7 (9)	C3—C2—C7—C6	0.9 (3)
F1'—C1—C2—C3	−6.6 (5)	C1—C2—C7—C6	−179.94 (18)
F3—C1—C2—C3	−23.5 (4)	C5—C6—C7—C2	−0.3 (3)
F2'—C1—C2—C3	−119.9 (4)	C6—C5—C8—C9	−1(5)
F1—C1—C2—C3	87.1 (3)	C4—C5—C8—C9	178 (100)
F2—C1—C2—C7	26.0 (8)	C5—C8—C9—C10	85 (5)
F3'—C1—C2—C7	−54.5 (10)	C8—C9—C10—C11	−11 (3)
F1'—C1—C2—C7	174.2 (5)	C8—C9—C10—C12	169 (3)
F3—C1—C2—C7	157.3 (4)	C12—C10—C11—C12^i^	−0.4 (3)
F2'—C1—C2—C7	60.9 (4)	C9—C10—C11—C12^i^	179.90 (16)
F1—C1—C2—C7	−92.1 (3)	C11—C10—C12—O1	−179.04 (15)
C7—C2—C3—C4	−0.8 (3)	C9—C10—C12—O1	0.6 (2)
C1—C2—C3—C4	−179.95 (18)	C11—C10—C12—C11^i^	0.4 (3)
C2—C3—C4—C5	0.0 (3)	C9—C10—C12—C11^i^	−179.92 (16)
C3—C4—C5—C6	0.6 (3)	C11^i^—C12—O1—C13	−1.1 (3)
C3—C4—C5—C8	−178.37 (17)	C10—C12—O1—C13	178.26 (17)
C4—C5—C6—C7	−0.5 (3)		

Symmetry codes: (i) −*x*, −*y*+2, −*z*+2.
